# Deep Learning-Based Biomimetic Identification Method for Mask Wearing Standardization

**DOI:** 10.3390/biomimetics9090563

**Published:** 2024-09-18

**Authors:** Bin Yan, Xiameng Li, Wenhui Yan

**Affiliations:** 1 College of Automation and Information Engineering, Xi’an University of Technology, Xi’an 710048, China; 2 College of Mechanical and Electronic Engineering, Northwest A&F University, Yangling 712100, China; 3 Shaanxi Key Laboratory of Complex System Control and Intelligent Information Processing, Xi’an University of Technology, Xi’an 710048, China; 4 Faculty of Liberal Arts, Northwest University, Xi’an 710127, China; 5 College of Mechanical Engineering, Xi’an Shiyou University, Xi’an 710065, China

**Keywords:** artificial intelligence, se module, BottleneckCSP, HSV space, mask, post-COVID-19 era

## Abstract

Deep learning technology can automatically learn features from large amounts of data, with powerful feature extraction and pattern recognition capabilities, thereby improving the accuracy and efficiency of object detection. [The objective of this study]: In order to improve the accuracy and speed of mask wearing deep learning detection models in the post pandemic era, the [Problem this study aimed to resolve] was based on the fact that no research work has been reported on standardized detection models for mask wearing with detecting nose targets specially. [The topic and method of this study]: A mask wearing normalization detection model (towards the wearing style exposing the nose to outside, which is the most obvious characteristic of non-normalized style) based on improved YOLOv5s (You Only Look Once v5s is an object detection network model) was proposed. [The improved method of the proposed model]: The improvement design work of the detection model mainly includes (1) the BottleneckCSP (abbreviation of Bottleneck Cross Stage Partial) module was improved to a BottleneckCSP-MASK (abbreviation of Bottleneck Cross Stage Partial-MASK) module, which was utilized to replace the BottleneckCSP module in the backbone architecture of the original YOLOv5s model, which reduced the weight parameters’ number of the YOLOv5s model while ensuring the feature extraction effect of the bonding fusion module. (2) An SE module was inserted into the proposed improved model, and the bonding fusion layer in the original YOLOv5s model was improved for better extraction of the features of mask and nose targets. [Results and validation]: The experimental results indicated that, towards different people and complex backgrounds, the proposed mask wearing normalization detection model can effectively detect whether people are wearing masks and whether they are wearing masks in a normalized manner. The overall detection accuracy was 99.3% and the average detection speed was 0.014 s/pic. Contrasted with original YOLOv5s, v5m, and v5l models, the detection results for two types of target objects on the test set indicated that the mAP of the improved model increased by 0.5%, 0.49%, and 0.52%, respectively, and the size of the proposed model compressed by 10% compared to original v5s model. The designed model can achieve precise identification for mask wearing behaviors of people, including not wearing a mask, normalized wearing, and wearing a mask non-normalized.

## 1. Introduction

The COVID-19 pandemic, which has just spread around the world, caused serious harm to health and property of 7 billion people worldwide. Although we have entered the post-pandemic era, potential risks of infectious diseases still exist at any time in the future, threatening human life and health safety.

Based on the transmission characteristics of respiratory infectious diseases and the actual situation of a global pandemic caused by them, citizens in many countries are required to wear masks when taking public transportation (buses, trains, subways, airplanes, etc.) and in places where people gather (shopping malls, schools, hospitals, agricultural markets, etc.). The main method used to determine whether citizens are wearing masks is manual inspection, which involves stationing inspectors at fixed entrances and exits. However, this method does not have the characteristic of being around the clock and is prone to missing detections in areas with a high population flow, which may pose safety risks to epidemic prevention and control. Based on the above reality, many scholars have studied vision-based mask detection algorithms.

On the other hand, due to the advantages of deep learning technology [1,2,3,4] in better mining and extracting the characteristics of collected data, it has been widely applied in the field of target detection in recent years [5,6]. In terms of deep learning-based mask wearing detection, up until now, there have been many deep learning networks which have been successfully utilized to detect mask targets, such as SCRNet [7], SSD [8], YOLOv2 [9,10], ResNet-50 [10,11], SSDMNV2 [9], VGG-16 [12,13], ResNet50 [10,11], MobileNetV2 [14], YOLOv3 [13,15], VGG-19 [16], Fast RCNN [17], YOLOv4 [18], etc. The relevant research in recent years is shown in Table 1. The commonality of these studies is that they are all based on deep learning techniques to detect mask wearing behavior. On the other hand, we analyzed and elaborated whether the models proposed by these research studies included detection functions for mask wearing standardization. The fact that our proposed detection model can not only detect whether people are wearing masks, but also further detect the standardization of the way people wear masks is also an advantage of the model proposed in this study.

**Figure 1:**
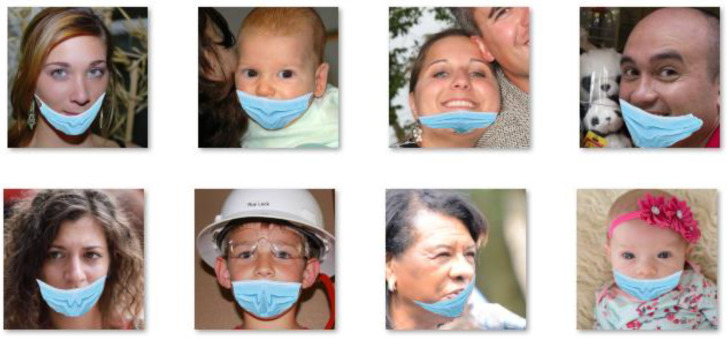
Situations of people wearing masks non-normalized.

Throughout the current research status of deep learning-based mask target detection algorithms, most existing algorithms only realized the judgment of whether people are wearing a mask by identifying a mask target, without precise detection of mask wearing standardization. However, in real life, the situation exists where people wear masks in a non-normalized way (as shown in Figure 1). It is obviously that the wearing style exposing the nose to outside is the most apparent and common characteristic of non-normalized styles. Therefore, the wearing behavior without the nose is covered is defined as non-normalized mask wearing. Since for many viruses that seriously threaten human life and health, such as COVID-19, one of their significant transmission ways is via the respiratory tract, potential safety hazards exist when only protecting the mouth utilizing a mask. The exposure of the nose to the outside is a great safety hazard, since the human body can also be infected after the virus invades via this route.

On the other hand, based on the analysis of the current research status in deep learning-based mask detection algorithms in Table 1, although a few existing methods realized detection of the standardization of mask wearing, the nose target itself were not identified individually, therefore the accuracy of mask wearing normalization detection needs to be further improved.

However, no research work has been reported on standardized detection algorithms for mask wearing with detecting nose targets specially. Therefore, in order to improve the accuracy and speed of mask wearing normalization detection, a mask wearing standardization detection method was proposed in this study based on artificial intelligence algorithm, aiming to achieve accurate identification of ‘not wearing mask’, ‘normalized wearing’, and ‘wearing mask non-normalized’. This study will provide technical support for the development of intelligent precision detection systems, reducing medical safety hazards and ensuring the safety of public life and health.

## 2. Materials and Methods

### 2.1. Improvement of YOLOv5s Network Architecture

#### 2.1.1. YOLOv5s Network Architecture

The principle of YOLOv5 is based on the convolutional neural network (CNN) in deep learning technology. CNN is a special type of neural network that can automatically extract features from images and use these features for recognition and classification. YOLOv5 uses a method called ‘single pass detection’ to identify objects in an image, meaning that the entire image only needs to be passed forward once to obtain the positions and categories of all objects. This makes YOLOv5 perform well in handling real-time object detection tasks in complex scenes. The advantages of the YOLOv5 network architecture are high detection accuracy and fast speed, with a maximum detection speed of 140 frames per second [20]. On the other hand, the weight of this model is small, nearly 90% smaller compared to the YOLOv4 model, which indicates that the YOLOv5 model is suitable to be deployed on embedded devices for real-time detection of targets. Since the detection accuracy, real-time performance, and whether the model is lightweight are directly related to the accuracy and efficiency of the detection, the YOLOv5 architecture was utilized and improved in this study to design a mask wearing normalization detection network.

The YOLOv5 network [21] specifically includes four architectures: YOLOv5s, YOLOv5m, YOLOv5l, and YOLOv5x. The main difference is the number of feature extraction modules and convolution kernels set in specific positions of the network, and the parameter quantity and volume of the four models increase sequentially. The detailed indicator parameters are shown in Table 2.

**Figure 2:**
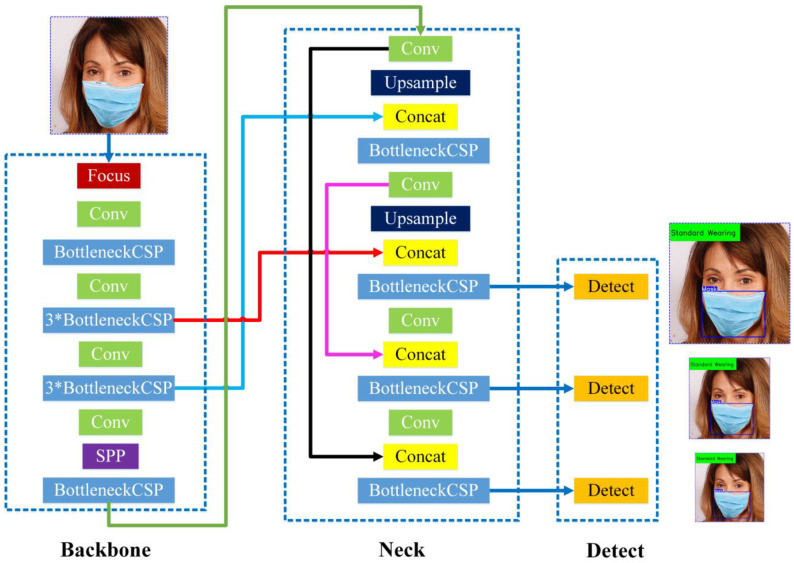
Architecture of the original YOLOv5s network.

Compared with the other three models, the YOLOv5s model has the fewest layers, the smallest volume, and the lowest number of parameters. At the same time, it can satisfy the recognition requirements for mask and nose for two types of target objects in detection tasks. In order to make the obtained detection model more suitable for deployment in hardware devices with different configurations of detection systems, a lightweight model with a small volume should be selected as often as possible. Therefore, since the requirements for real-time detection and a lightweight model are overall consideration of accuracy, efficiency, and volume of the model, the design improvement of a mask wearing normalization detection network was planned to be realized in the YOLOv5s architecture (Figure 2).

**Figure 3:**
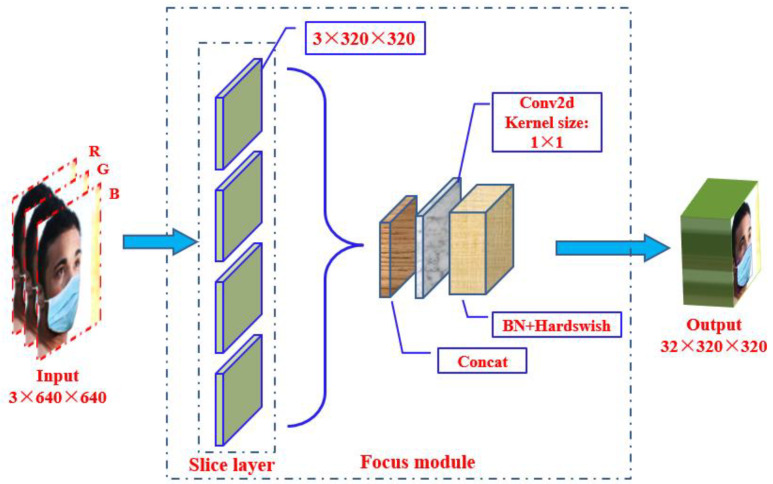
Focus module.

The YOLOv5s architecture mainly consists of three components, including a backbone network, a neck network, and a detect network. The backbone network is a convolutional neural network that aggregates different fine-grained images and forms image features. Specifically, the first layer of the YOLOv5 backbone network is a focus module (Figure 3), which is designed to reduce the calculations of the model and accelerate the training speed. The functions of the focus module are as follows: Firstly, use a slicing operation to divide the input 3-channel image (YOLOv5s’ default input image size is 3 × 640 × 640) into four slices of 3 × 320 × 320 each. Secondly, use the concat operation to deeply connect the four parts; the size of the output feature map is 12 × 320 × 320. Then, through a convolutional layer composed of 32 convolution kernels, the size of the output feature map is 32 × 320 × 320. Finally, the results are output to the next layer through a BN layer (batch normalization) and a Hardswift activation function.

The main function of the Focus module in YOLOv5 is to improve the model’s ability to detect small targets. The focus structure is a special layer that enhances the model’s detection performance for small targets through specific operations. The design of this structure aims to solve the problem of small targets being difficult to detect in object detection tasks, as the size of small targets is small and their features are not obvious. The structure downsamples the input feature map by adding a convolutional layer before the input layer, and then upsamples feature map through another convolutional layer, thereby making the model more focused on features of small targets and improving detection performance. The utility of this structure can improve the accuracy and recall of the model, especially in detection of small objects such as nose targets, which has significant advantages.

**Figure 4:**
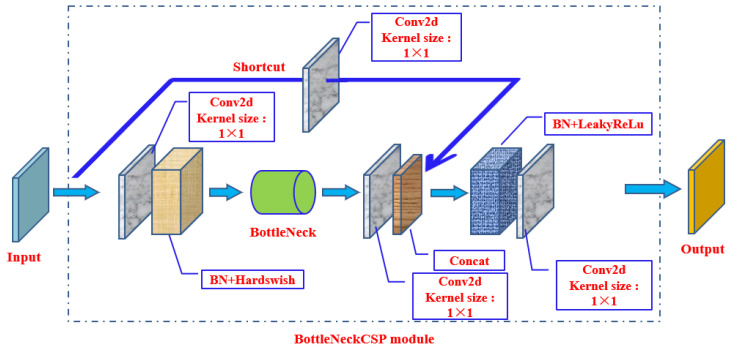
BottleneckCSP module.

**Figure 5:**
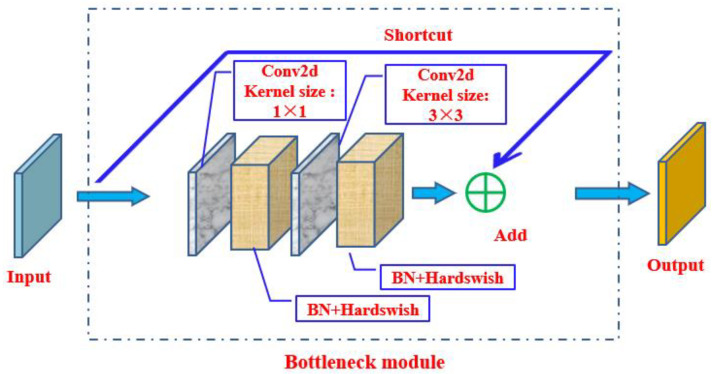
Bottleneck module.

The BottleneckCSP module (Figure 4) is the third layer of the backbone architecture, designed to better extract deep features of images. The BottleneckCSP module mainly consists of a Bottleneck module (Figure 5), which is a residual architecture that connects a convolutional layer with a kernel size of 1 × 1 (Conv2d + BN + Hardswift activation function) with another convolutional layer with a kernel size of 3 × 3. The output of the Bottleneck module is the sum of the output of that part and the initial input through the residual architecture.

The core function of the Bottleneck layer in YOLOv5 is mainly to reduce computational complexity and enhance feature expression ability through specific structural design. This layer structure optimizes the information flow path in the network, enabling the model to maintain high performance while reducing the consumption of computing resources.

The BottleneckCSP structure uses a concat operation to enable model to learn more features. The BottleneckCSP structure divides the original input into two branches, performs convolution operations separately to reduce the number of channels by half, and finally merges two branches through the concat operation to make the input and output of the BottleneckCSP the same size. This design is intended to enable the model to learn more features, improving its performance and accuracy. Through the concat operation, different levels of feature maps are fused, preserving and accumulating more feature information from different receptive fields, thus obtaining feature maps with rich information, enabling the recognition of masks and nose targets in various scales.

The input of the BottleneckCSP module is imported into two branches, and the number of channels in the feature map is halved through a convolution operation. Then, using the bottleneck architecture and Conv2d layer in branch two, the outputs of branch one and branch two are concatenated to achieve deep connections. Finally, the output feature map of the module is obtained through BN and Conv2d layers. In addition, the size of the output feature map is the same as the input size of the BottleneckCSP.

**Figure 6:**
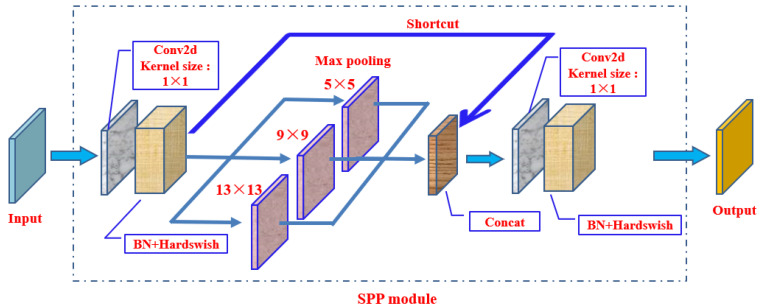
SPP module.

The ninth layer of the backbone network is the SPP module (Spatial Pyramid Pooling) (Figure 6), which aims to improve the model’s receptive field by converting any size feature map into a fixed size feature vector. The input feature map size of the SPP module is 512 × 20 × 20. Firstly, a convolutional layer with a kernel size of 1 × 1 is used to output a feature map with a size of 256 × 20 × 20. Secondly, the feature map is connected in depth with the output map subsampled through three parallel max pooling layers, resulting in an output feature map size of 1024 × 20 × 20. Finally, a 512 × 20 × 20 output feature map was obtained through convolutional layers.

The main function of the SPP module in YOLOv5 is to achieve fusion of local and global features. The design purpose of the SPP module is to ensure that feature vectors between fully connected layers are of fixed size. Compared with conventional methods, SPP avoids the problems that may arise from cropping and deformation operations by unfolding different feature maps into fixed size feature vectors. This feature enables the SPP module to ignore input size and generate fixed length outputs, thereby improving the recognition accuracy of the network. In addition, by utilizing different pooling kernel sizes to extract features, SPP can obtain rich feature information, which is beneficial for improving the recognition ability of the network. In YOLOv5, the role of the SPP module is not limited to feature extraction and dimensionality reduction. It also emphasizes the fusion of local and global features, which helps improve the model’s discriminative ability and capture useful information, thereby enhancing the accuracy of mask and nose detection.

The neck architecture is a series of feature aggregation layers with mixed and combined image features, which adopts a new FPN structure (feature pyramid network) to enhance the bottom-up path. Therefore, the transmission of low-level features has been improved, enhancing the detection of objects at different scales. Therefore, it is possible to accurately detect the same target object with different sizes.

The detection architecture is mainly used for the final detection part of the model, which applies anchor boxes on the feature map output by the previous layer and outputs a vector containing the class probability, object score, and position information of the bounding boxes around the target object. The detection architecture of YOLOv5s consists of three detection layers, whose inputs are feature maps of 80 × 80, 40 × 40, and 20 × 20, used to detect objects of different sizes. Each detection layer ultimately outputs a 21-channel vector (3 anchor boxes x (2 categories + 4 surround box position coordinates + 1 category probability)), and then labels the predicted bounding boxes and target categories to achieve object detection in the image.

#### 2.1.2. Improvement of Backbone Architecture

In order to compress the volume of the algorithm model as much as possible while ensuring detection accuracy, based on the YOLOv5s architecture, the backbone network was optimized and improved in this study for facilitating its deployment in hardware devices at a later stage. Therefore, based on effectively extracting the features of target objects in the image to ensure detection accuracy, the weight parameter quantity and volume of the architecture were reduced, in order to realize the lightweight and improved design of the mask wearing normalization detection model.

Since the target to be detected is composed of two classes, the requirement for a deeper network structure to extract its features is not essential. In order to reduce the weight parameter quantity of the network while ensuring the feature extraction effect of the bonding fusion module as much as possible, the improvement strategy of this study is to remove the convolution module on the bonding fusion layer and main branch channels of the original BottleneckCSP module. The strategy achieves the fusion of lower-level feature maps with higher resolution, containing more target object position and detail information, and high-level feature maps with stronger semantic information after multiple feature extraction modules.

**Figure 7:**
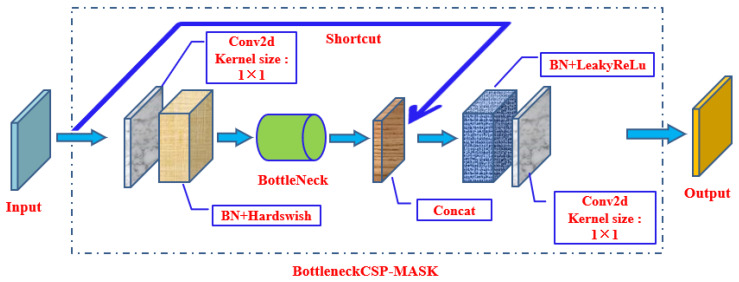
BottleneckCSP-MASK module (improved BottleneckCSP module).

The improved BottleneckCSP module is shown in Figure 7 and named BottleneckCSP-MASK. Since the targets that need to be detected include two kinds of objects, nose and mask, with relatively low complexity, in order to implement the light weight of the designed network, the parameter count of convolutional layers was reduced: the Conv2d module in the shortcut of the original BottleneckCSP was removed, and the Conv2d module in the main branch of the original BottleneckCSP was also removed, making the improved network more suitable for hardware applications in identification systems to achieve real-time detection. On the other hand, to reduce the size of the detection model and the overall parameter count of backbone network, achieving a light weight of the model, in four places in the original backbone network where the BottleneckCSP modules were originally used, they were replaced with four connected BottleneckCSP-MASK modules instead.

#### 2.1.3. Inserted Design of SE Module in Backbone Network

Due to the differences in the appearance of masks and noses compared to background objects in images, in order to improve the detection accuracy of mask wearing normalization, the attention mechanism [22] in machine vision was inserted into the architecture of the mask detection network to better extract the features of two types of target objects in this study.

**Figure 8:**
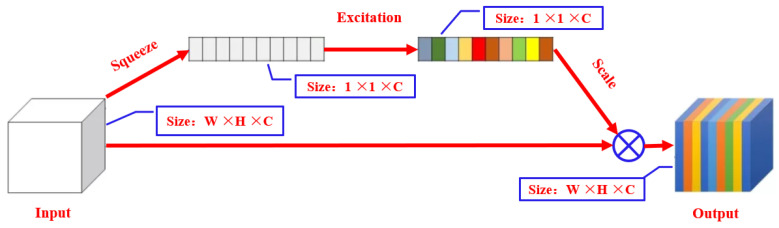
Architecture of SE module.

The SE module (Squeeze and Network, SENET) [23,24] is a visual attention mechanism architecture in which a new feature recalibration strategy is automatically obtained through learning, indicating the importance of each feature channel, and then promoting useful features while correspondingly suppressing unnecessary features. Due to the small computational complexity of the SE module, it can effectively improve the expression ability of the model and optimize the learning content. Therefore, this study inserts it into the backbone of the improved YOLOv5s network to improve the detection accuracy of the model. The schematic diagram of the SE module structure is shown in Figure 8. This study embeds it into the 5th, 7th, 11th, and 14th layers of the improved backbone architecture.

SE modules were embedded in 5th, 7th, 11th, and 14th layers of the improved backbone network in this study. The embedding in the 7th layer: The first detection layer (Detect layer) of the improved YOLOv5s architecture can achieve recognition of relatively large target objects in the image. In the improved network architecture, the feature map output from the 7th layer is concatenated with the high-level feature map output from the 20th layer and input to the first Detect layer. Therefore, an SE module was embedded at the position of the 7th layer, in the front end, so that the feature map containing the high-dimensional feature information output from the 20th layer was fused with the feature map highlighting the information of larger target objects in the 7th layer through the SE module, which is conducive to the accurate recognition of relatively larger target objects in images by the first detection layer.

Embedding in the 14th layer: The SPP module in the 13th layer of the improved network achieves the fusion of local and global features, enriching the expressive power of the feature map and facilitating the recognition of large differences in target size in the image. Afterwards, the SE module was embedded to further enhance the output of obvious features in the SPP module feature map and suppress unimportant features.

In the improved YOLOv5s backbone network, there are multiple feature extraction modules with convolution operations, such as the Bottleneck CSP-MASK module and Conv module, both of which have the function of extracting image features. Therefore, by integrating the SE module after these feature extraction modules, it is possible to mine the interrelationships between channels in the deep feature map, extract detailed information, and further optimize the features extracted through the convolutional layer based on the deep feature map obtained. Therefore, based on the above improvements, SE modules were embedded between layers 1–7 and 7–14 of the improved backbone network in this study.

Embedding in the 5th layer: As the degree of feature extraction by the backbone network increases with the number of layers, if the front of the backbone network is not fully capable of feature extraction, embedding SE modules at excessively high positions for feature optimization is not effective. On the other hand, an architecture where two SE modules are connected can cause resource redundancy, so the SE module was embedded into the fifth layer of the improved backbone network in this study.

**Figure 9:**
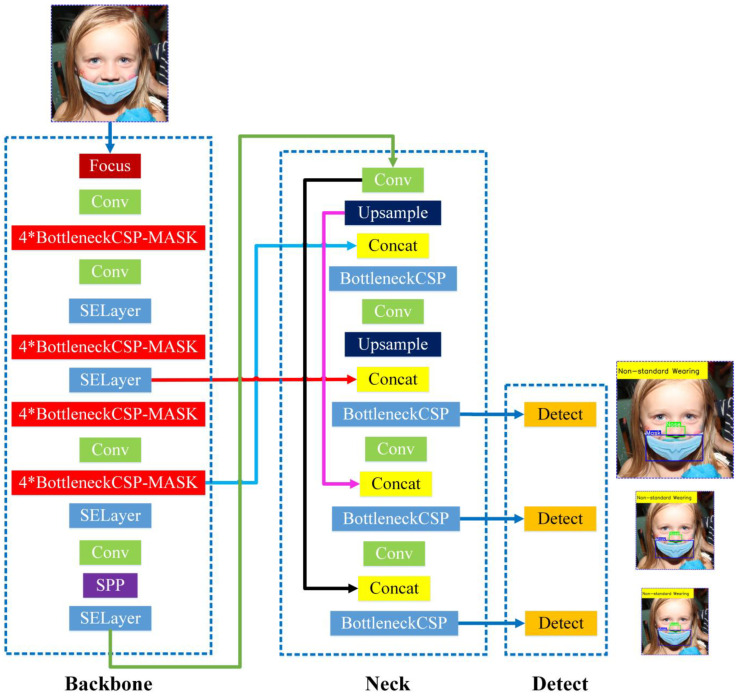
Architecture of YOLOv5s-MASK network.

Embedding in Layer 11: In order to perform cross-fusion between low-level features and higher-level feature maps containing strong semantic information to enhance the network’s feature extraction capability, this study determined to embed the SE module between layers 7–14 into the 11th layer after the Bottleneck CSP-MASK module. The improved YOLOv5s architecture is shown in Figure 9.

#### 2.1.4. Improvement of Fusion Feature Layer

Integrating feature maps of different scales is an important way to improve model detection performance. The purpose of feature fusion is to combine the extracted features from an image into features with stronger discriminative ability compared to the input features. Low-level feature maps have higher resolution and contain more positions and detailed information on target objects. However, due to the limited number of features extracted through convolutional layers, low-level feature maps have lower semantics and contain more noise. Advanced feature maps have rich semantic information, but their resolution is low and their ability to perceive image details is relatively insufficient. Therefore, effectively integrating high-level and low-level features is the key to improving model detection performance.

Based on the improved design of the YOLOv5s backbone architecture in Section 2.1.2 and Section 2.1.3, and the size of the output feature maps of each layer in the improved network, the original YOLOv5s architecture’s bonding fusion lines of 5th layer and 17th layer (red bonding fusion line in Figure 2), 7th layer and 13th layer (light blue bonding fusion line in Figure 2), 11th layer and 23rd layer (black bonding fusion line in Figure 2) were, respectively, changed to the bonding fusion lines of the 7th and 21th layers (red bonding fusion line in Figure 9), 10th and 17th layers (light blue bonding fusion line in Figure 9), 15th and 27th layers (black bonding fusion line in Figure 9) of the improved network in this study. The designed mask wearing normalization detection network architecture (named YOLOv5s-MASK) is shown in Figure 9.

The relation to its role in mask detection of each component of the designed YOLOv5 network is explained as follows: The function of the Backbone layer is to extract the features of mask and nose targets from the original image, and process the input image through a series of convolutional and pooling layers, gradually reducing the size of the feature map while increasing the number of channels, with the aim of preserving and extracting important features from the image. The feature maps extracted through the backbone will be passed on to the subsequent Neck and Detect layers for processing. The Neck layer is the network structure between the Backbone layer and the Detect layer. The main function of this layer is to further fuse and upsample the features extracted by the Backbone network, in order to provide higher-level semantic information and enhance the model’s detection ability for images of different scales. The Detect layer is responsible for mask wearing normalization detection and recognition. The Detect layer fuses and transforms features of different scales, which helps to obtain higher-level semantic information and contextual relationships, further process the extracted features, and generate the final mask and nose detection results.

**Figure 10:**
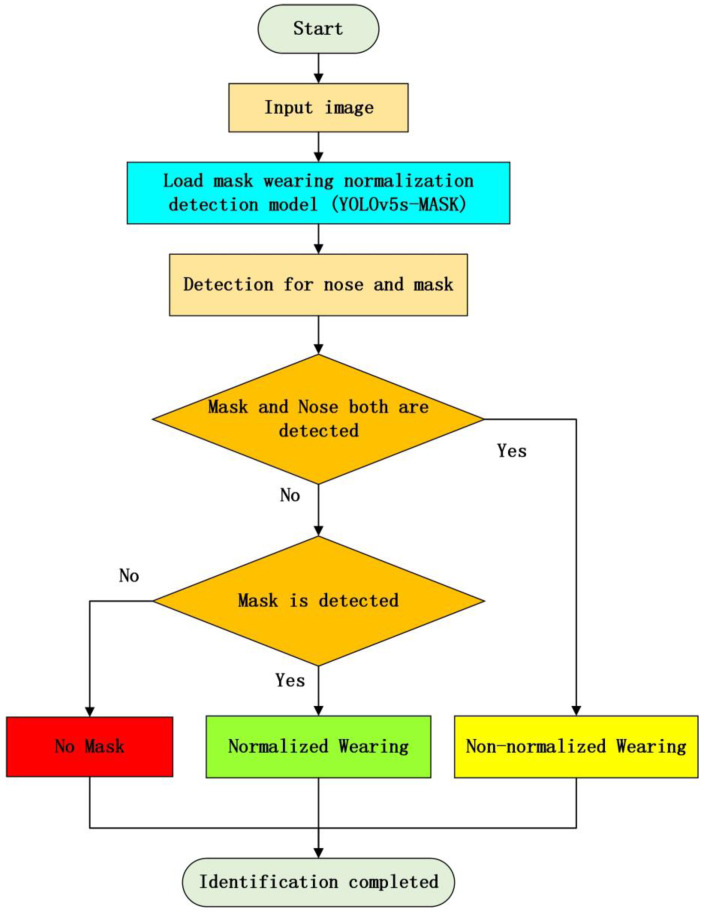
Overall flowchart of the proposed mask wearing normalization detection method.

Furthermore, the overall flowchart of the proposed mask wearing normalization detection method is shown in Figure 10 as follows.

### 2.2. Acquisition and Preprocess of Image Data

#### 2.2.1. Image Data Acquisition Methods

The explanation of the training data for the proposed deep learning model in the article is because the training data have a significant impact on the detection performance of the final model. The main reasons why deep learning models require a large amount of data for training are to improve their generalization ability, accuracy, and robustness. Deep learning models train to learn task-related features and representations, adjust weights and parameters, and automatically learn how to extract useful features from input data, which are crucial for the successful execution of tasks. By learning with a large amount of training data, the model is able to capture general patterns in the data, rather than just remembering specific samples in the training set, thus having generalization ability, that is, being able to make good predictions when faced with unseen data. Using the dataset described in the article to train the model, the deep learning model obtained after training can not only detect the data in the article well, but also detect data in various other uncertain scenarios. Therefore, the detection scope of the proposed model is not limited to the dataset mentioned in the article.

**Figure 11:**
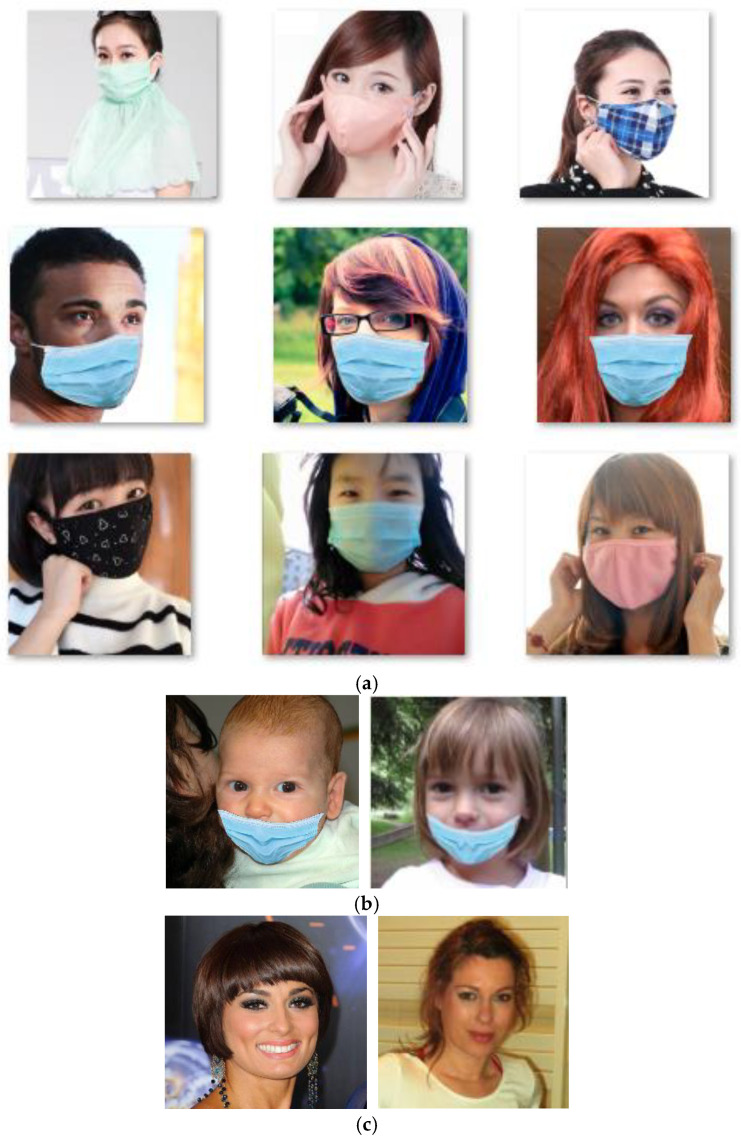
Examples of people wearing masks normalized (**a**), wearing masks non-normalized (**b**), and not wearing masks (**c**) in datasets.

The research aim of this study is to achieve precise detection of mask wearing normalization in the post-pandemic era. The original data were obtained on the internet, which mainly includes men and women of different ages wearing masks and without masks in different scenes. On the other hand, based on widely used mask styles, the ‘non-normalized wearing’ mask category data were independently prepared from batch images of people not wearing masks. The original dataset included three types of data: the images of people who do not wear mask (not wearing mask), the images of people who wear masks in the standard way (normalized wearing), and the images of people who wear masks in a non-standard way (wearing mask non-normalized). Examples are shown in Figure 11:

#### 2.2.2. Preprocessing of Images

More than 1500 images were obtained, some of which were difficult to distinguish manually due to their blurry image quality and could not be used for training deep learning detection models, so they were removed. Ultimately, 1466 valid image data were retained. The 1466 images with stable quality were randomly divided into a training set and a testing set, whereby we randomly selected 150 images (50 images for each category including not wearing mask, normalized wearing, and wearing mask non-normalized) for model testing.

Using an image data annotation software called ‘LabelImg’ (Version 1.8.1), we drew an external rectangular box around the target in the training set images to achieve manual annotation. The image is labeled based on the smallest rectangle around the target to ensure that the rectangle contains as little background area as possible. Based on the analysis of different mask wearing situations mentioned above, ‘mask’ and ‘nose’ labels were, respectively, marked for the mask and nose targets in the image. Finally, we generated the XML of the mat file after saving the comments.

In order to enrich the image data of the training set and better extract the features of various masks and noses, data augmentation was utilized on training set images. For improving the generalization ability of mask wearing normalization detection model, data augmentation was used to enhance and decrease the brightness of images in the training set.

The HSV (hue, saturation, value) color space is a model used to represent colors, based on the human perception of colors. The HSV color space describes colors through three dimensions: hue, saturation, and value. Hue: hue represents the basic type of color, usually expressed in terms of angle, ranging from 0° to 360°, corresponding to the visible spectrum of colors such as red, orange, yellow, green, cyan, blue, purple, etc. Saturation: saturation represents the purity or intensity of a color. The higher the saturation, the purer the color, and the lower the saturation, the closer the color is to gray. In the HSV model, saturation is typically expressed as a percentage, ranging from 0% (gray) to 100% (purest color). Value (value or brightness): brightness represents the degree of brightness or darkness of a color. The higher the brightness, the brighter the color, the lower the brightness, the darker the color. Brightness is usually expressed as a percentage, ranging from 0% (black) to 100% (white).

Image brightness adjustment based on the HSV color space was used to augment the training data and enhance the generalization performance of the model in this study. The purpose is to generate image data with different brightness levels.

**Figure 12:**
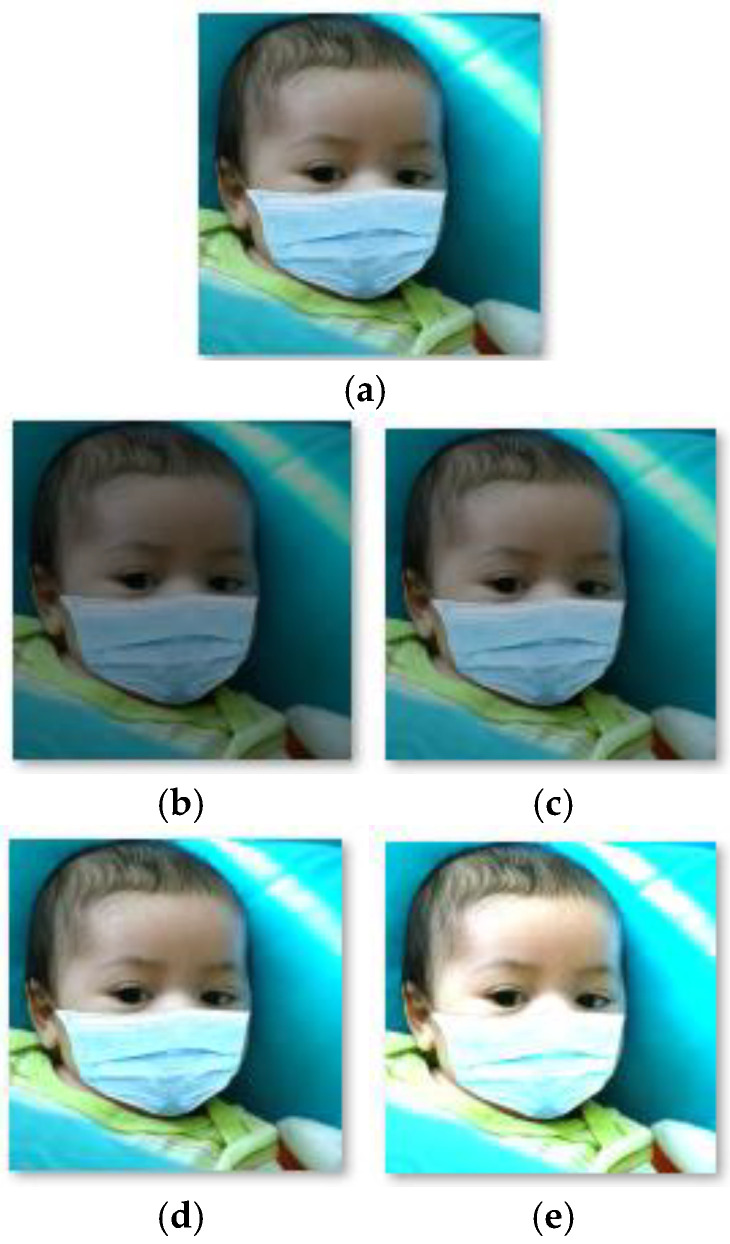
The original example image (**a**) and the resulting images after enhancement of 0.6 × V (**b**), 0.8 × V (**c**), 1.2 × V (**d**), and 1.6 × V (**e**).

Firstly, the ‘rgb2HSV’ function was used to convert the original image into HSV space. Secondly, the V component (brightness component) of the image was multiplied by different coefficients. Finally, the synthesized HSV space image was converted to the RGB space using the ‘hsv2rgb’ function, achieving brightness enhancement and reduction of the image. In this study, two brightness intensities were generated using brightness enhancement, including (H + S + 1.2 × V) and (H + S + 1.6 × V). In addition, reducing brightness can generate two other brightness intensities, including (H + S + 0.6 × V) and (H + S + 0.8 × V). An enhanced example image is shown in Figure 12.

A total of 1466 images obtained after data augmentation were used as training set data for training of the mask wearing normalization detection model. The specific quantity distribution of different image categories in the training and testing sets is shown in Table 3 below.

### 2.3. Network Training

#### 2.3.1. Training Platform

Based on a Lenovo Legion Y7000P (Lenovo (Beijing) Co., Ltd., Beijing, China) computer (NVIDIA Geforce RTX 2060 GPU, Intel (R) Core (TM) I7-9750H CPU, 2.6 GHz, 16 GB memory; 6 GB video memory), the PyTorch deep learning framework was established on the Windows 10 operating system in this study, and Python language was used to write the program code and to call Cudnn, CUDA, OpenCV, and other required libraries, to realize the training and testing of the mask wearing normalization detection model.

In this study, the detailed information on hyperparameter tuning are as follows: the designed YOLOv5s-MASK network was trained by the stochastic gradient descent (SGD) optimization strategy utilizing an end-to-end way. The initial learning rate was set as 0.01. The batch size of model training was 4, and regularization was done by the BN layer to update the weight of the model each time. The number of training epochs was 100. The final detection model is trained based on the above parameter settings. After training, the weight file of the detection model obtained was saved, and the test set was used to evaluate the performance of the model.

**Figure 13:**
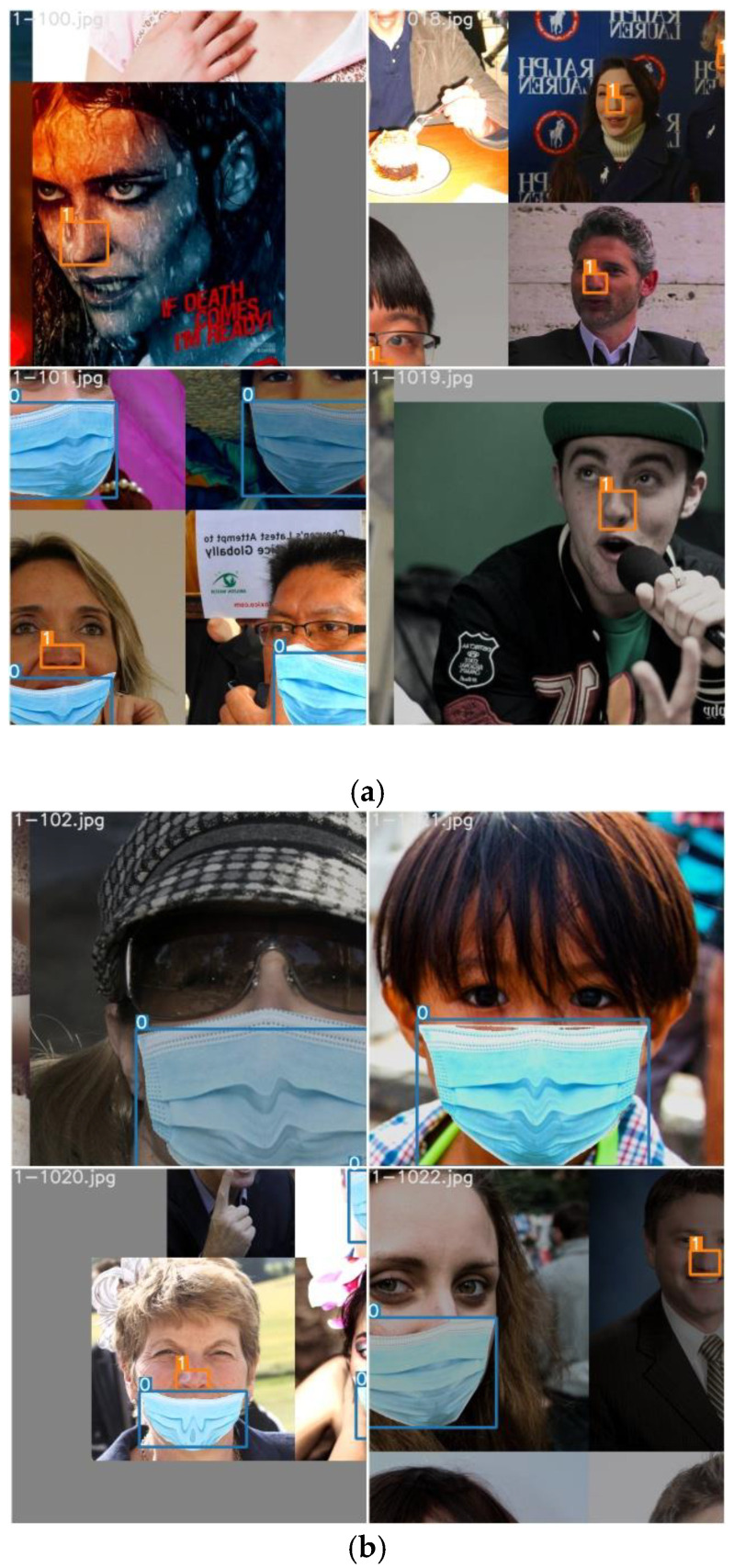
Two examples (**a**,**b**) of generated training images utilizing Mosaic method.

In this study, the improved YOLOv5 network was trained using the Mosaic data augmentation method, which was mainly used to improve the model’s detection performance for small targets and accelerate the training speed. Mosaic data augmentation is a data augmentation technique which concatenates four images by randomly scaling, cropping, and arranging them to generate a new training image. The advantage of this method is to improve the detection performance for small targets: by stitching multiple images together, the frequency of small targets appearing in the training images can be increased, allowing the model to better learn the features of small targets during training process, thereby improving its detection ability for small targets such as nose and a mask. Accelerated training speed: Due to the ability of the Mosaic data augmentation method to generate more training samples, it effectively increases the diversity of the dataset, which helps the model converge faster and thus shortens training time. Therefore, the generalization ability of the model can be guaranteed. Examples of generated training images utilizing the Mosaic method are shown in Figure 13.

Data augmentation techniques were utilized on training set images during hyperparameter settings for model training in this study, including image flipping operations, image translation, and image scale transformation, to enhance the robustness of the model against different image variations.

#### 2.3.2. Training Results

**Figure 14:**
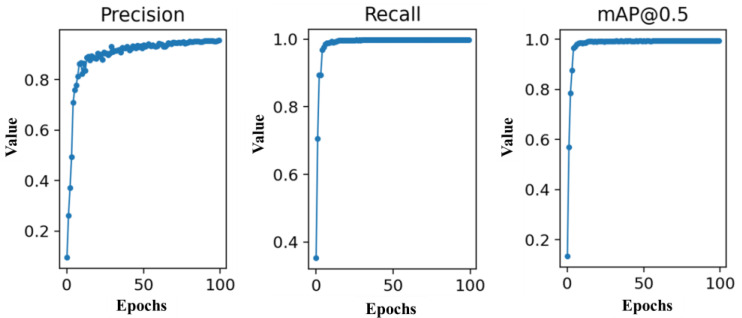
Variation curves of precision, recall, and mAP indicators in model training.

**Figure 15:**
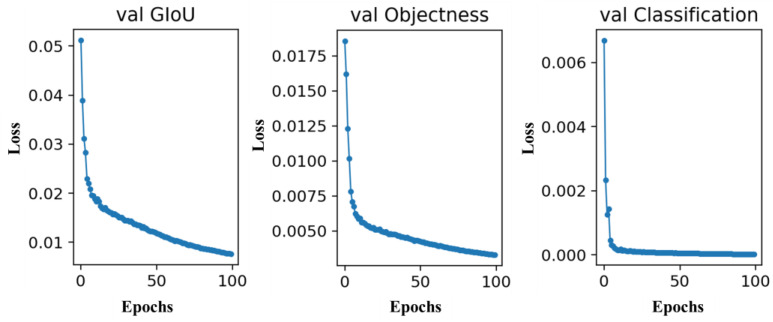
Network training loss curve.

The variation curves of precision, recall, and mAP indicators in model training are shown in Figure 14. The boundary box localization loss (val GIou loss), target object loss (val Objectivity loss), and class classification loss (val Classification loss) curves during the network model training validation stage are shown in Figure 15. According to the figures, the results indicated that the loss value of the network rapidly decreased during the first 50 epochs of training. After 75 epochs of training, the loss value tended to stabilize. Therefore, the model output after 100 epochs of training was utilized as the mask wearing normalization detection model in this study.

### 2.4. Test and Evaluation of Model

#### 2.4.1. Evaluation Indicators of Target Object Detection Performance

Objective evaluation indicators such as precision (*Precision_i_*, *P_i_*) (1), recall (*Recall_i_*, *R_i_*) (2), average precision (*AP_i_*) (3), *F*1 Score (*F*1*_i_*) (4) and mean average precision (*mAP*) (5) were utilized to evaluate the detection performance of the trained model towards mask and nose targets in this study. The calculation equations are as follows:

(1) $${Precision}_{i}=\frac{TP_{i}}{TP_{i}+FP_{i}}$$

(2) $${Recall}_{i}=\frac{TP_{i}}{TP_{i}+FN_{i}}$$

(3) $$AP_{i}=\int_{0}^{1}p_{i}(R_{i})dR_{i}$$

(4) $$F1_{i}=\frac{2}{\frac{1}{{\mathrm{Precision}}_{i}}+\frac{1}{{\mathrm{Recall}}_{i}}}$$

(5) $$mAP=\frac{1}{2}\sum_{i=1}^{2}AP_{i}$$

In the formulas, the values of *i* can be 1 or 2 corresponding to two types of target objects to be detected. Specifically, *TP*_1_ and *TP*_2_ represent the number of correctly identified masks and noses. *FP*_1_ and *FP*_2_, respectively, represent the quantity of incorrectly identified masks and noses. *FN*_1_ and *FN*_2_, respectively, represent the number of unrecognized masks and noses.

#### 2.4.2. Evaluation Indicators of Mask Wearing Normalization Detection

The objective evaluation indicator *P_mask_* was used to evaluate the overall performance of the trained mask wearing normalization detection model in this study, and its calculation formula is as follows:

(6) $$P_{mask}=\frac{pic_{normalized}+pic_{non-normalized}+pic_{no\text{-}mask}}{pic_{all}}\times 100\%$$

In the formula, $pic_{normalized}$ represents the number of correctly recognized images with people practicing normalized wearing. $pic_{non-normalized}$ represents the number of correctly recognized images with people wearing mask non-normalized. $pic_{no\text{-}mask}$ represents the number of correctly recognized images with people not wearing masks. $pic_{all}$ is the amount of all images that need to be detected.

## 3. Results and Discussion

### 3.1. Results and Analysis of Mask Wearing Normalization Detection

In order to verify the performance of designed mask wearing normalization detection model, further analysis was conducted towards the detection results of model on test set images. There are a total of 201 detection targets in 150 test set images, including 101 nose targets and 100 mask targets. The specific recognition results of the model are shown in Table 4. This indicated that the precision, recall, and average accuracy of the proposed model for detecting nose and mask targets were 98%, 98%, and 98.5%, and 98%, 100%, and 99.5%, respectively. The overall precision, recall, and average precision were 98%, 99%, and 99%, respectively.

On the other hand, the test set contains a total of 150 images of three mask wearing styles. The detailed distribution for the number of different mask wearing styles is shown in Table 5. According to the table, the recognition accuracy of the model for the three categories normalized wearing, wearing mask non-normalized, and not wearing mask were 100%, 100%, and 98%, respectively. The overall detection accuracy (*P_mask_*) was 99.3%.

**Figure 16:**
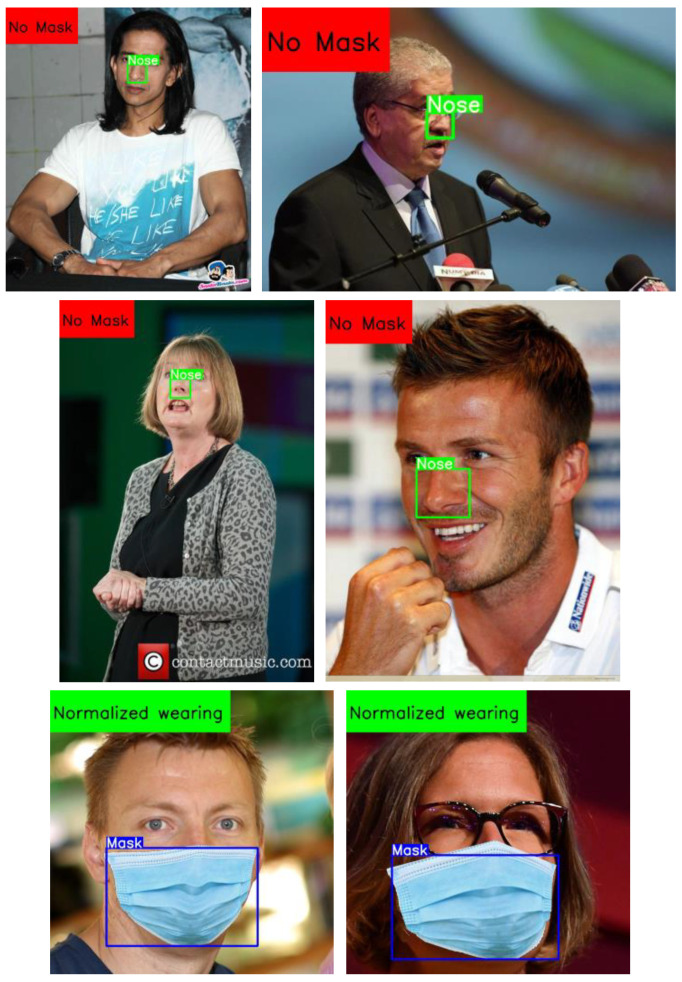

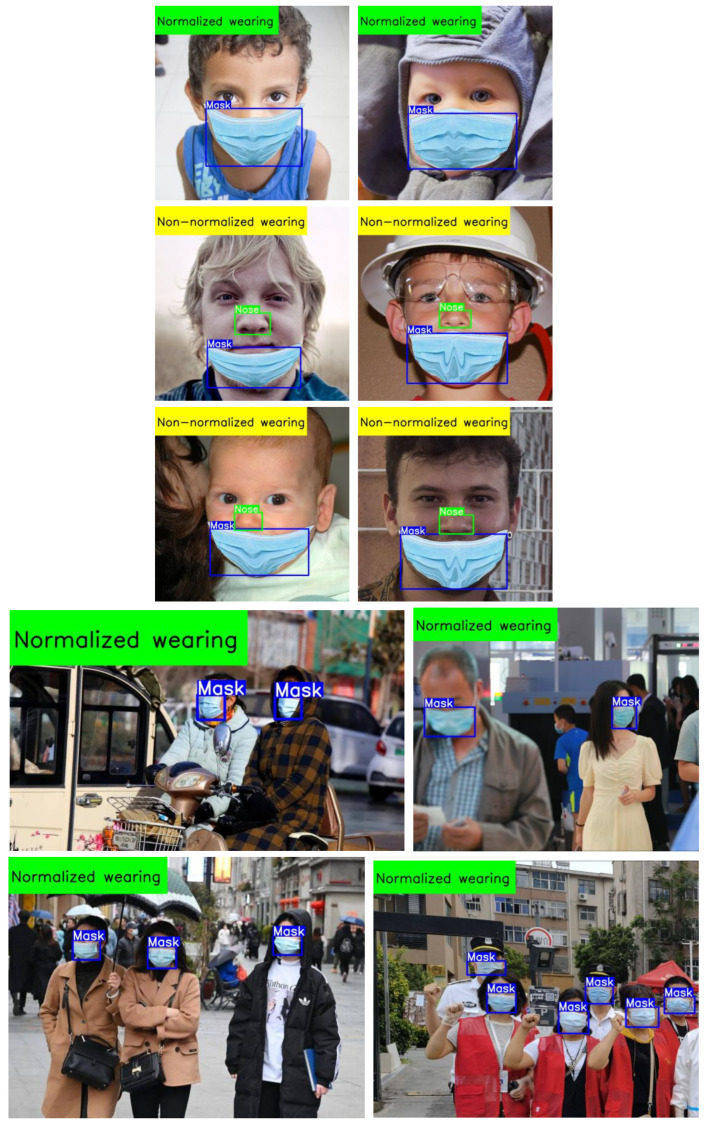

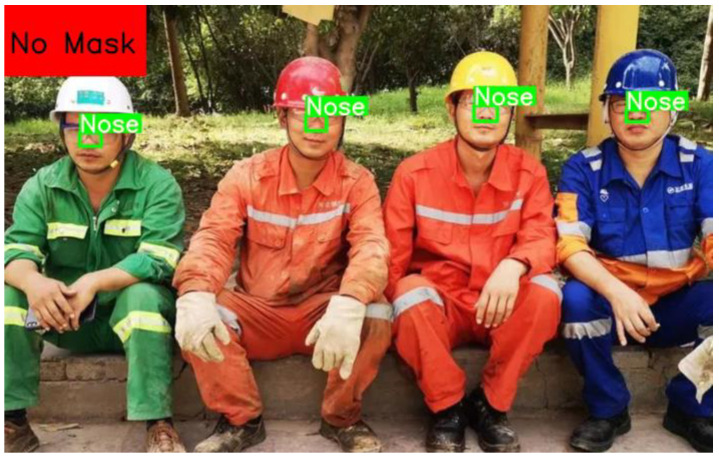
Recognition results for the three mask wearing styles not wearing mask, normalized wearing, and wearing mask non-normalized based on the YOLOv5s-MASK network.

Examples for detection results of the model proposed in this study for mask wearing normalization detection are shown in Figure 16. Among them, the identified nose and mask targets were marked with green and blue bounding boxes, respectively. The recognition results of normalized mask wearing were automatically displayed in the upper left corner of the resulting image. According to Figure 16, this indicated that the proposed recognition model could effectively identify whether people are wearing masks and whether they were wearing masks in a standardized style in images.

From Figure 16, it can be seen that the proposed detection algorithms can accurately detect mask and nose targets for different numbers of persons. On the other hand, the detection result images also show that the tested images contain different complex background objects, and the designed algorithm has robust performance in detecting target objects in complex backgrounds, indicating the effectiveness of the proposed method.

In this study, 1466 raw images were used for the training set and 150 images were used for the testing set. Although the number of images in the testing set was relatively small, the test set data cover various categories of mask wearing images, and the loss curve of model training and data analysis of the model performance testing results demonstrate the objectivity of the model performance evaluation. Therefore, the amount of data used for validation and testing in this study can meet the evaluation of model performance.

In order to analyze the impact of each component of the improved model on the overall performance, an ablation study was conducted in this study. The network performance of different models is shown in Table 6.

We conducted ablation experiments on four models, including YOLOv5s, YOLOv5s + SE, YOLOv5s + BottleneckCSP-MASK, and YOLOv5s + BottleneckCSP-MASK + SE, comparing and analyzing the average detection accuracy mAP values of different models on test set images. According to Table 6, it can be seen that embedding the SE visual attention mechanism module on the basis of the original YOLOv5 model improved the detection accuracy of the model for target objects. On the other hand, although the improved BottleneckCSP-MASK design has made lightweight improvements to the original BottleneckCSP module, embedding multiple BottleneckCSP-MASK modules at different positions in the model resulted in better mAP values than those of the original v5s model. The detection mAP value of the YOLOv5s + BottleneckCSP-MASK + SE network architecture proposed in this study was slightly higher than the other three models.

### 3.2. Comparison of Detection Results Utilizing Different Object Detection Models

In order to further analyze the recognition performance of the proposed algorithm for mask wearing normalization, the designed YOLOv5s-MASK network was compared with the original YOLOv5s, v5m, and v5l networks on the test set in this study. The detection accuracy and average detection time of target objects were taken as evaluation indicators. The recognition results including size and number of parameters for each network model are shown in Table 7.

According to Table 7, the mAP value of YOLOv5s-MASK recognition model proposed in this study was the highest. For the recognition speed of the model, the average detection speed of the YOLOv5s-MASK model was 0.014 s per image (71 fps) (fps, frames per second), which was 5.07 and 5.46 times the speed of the YOLOv5m and YOLOv5l networks, respectively.

On the other hand, it can be seen from Table 7 that the size of the proposed recognition model in this study was only 12.6 MB, accounting for 90% of the original YOLOv5s model. This indicated that the proposed model cannot only ensure the detection accuracy, but also realize the lightweight properties of the network effectively. Therefore, the designed model is more suitable for deployment on visual hardware devices. Although the recognition speed of the proposed model is slightly lower than that of the original YOLOv5s network, the average detection frame rate can reach 71 fps, which already satisfies the requirements of real-time detection for mask wearing normalization. On the other hand, if the model is too small or lightweight, it may lead to insufficient feature extraction of the target object, thereby affecting the accuracy of detection.

Compared with other algorithms, the proposed improved YOLOv5s detection model has a relatively small advantage in the detection accuracy of mask and nose targets. However, it is worth noting that the proposed model has potential advantages in terms of model size and parameter quantity. The parameter quantity of the model was reduced by one order of magnitude compared to the original YOLOv5s model, indicating that the proposed model is more suitable for deployment on hardware of medical and health monitoring systems for real-time detection tasks.

### 3.3. Comparison of the Proposed Model with Other Improved YOLOv5 for Object Detection

The consequences of natural disasters leave victims trapped in rubble, which is difficult for smart drones to detect. For victims with low visibility in harsh disaster environments and victims of various scales, to overcome the above challenges, a scale aware attention network based on transformer fusion (TFSANet) was proposed by robustly integrating the potential interactions between RGB and thermal images, and to address victim detection issues of various scales [25]. A transformer fusion model was developed to combine a dual-stream backbone network that effectively integrates the complementary characteristics between RGB and thermal images. Furthermore, a scale-aware attention mechanism was designed and embedded into the head network for adaptive adjustment to capture the receptive field sizes of victims at different scales.

Another model for detecting objects under difficult conditions has been proposed in a different study [26]. To create an environment, drones were used to capture images in the situations where detecting objects of various heights, weather, and backgrounds can be confusing. In addition, it aims to detect objects in these environments and improve detection performance.

Another study [27] proposed a marine biological target detection architecture based on an improved YOLOv5 framework, since there are challenges in identifying underwater organisms, such as decreased image quality, complex backgrounds, and detecting marine organisms at different scales. To address these issues and further improve the accuracy of related models, firstly, the backbone framework of the Real Time Object Detection Model (RTMSet) was introduced. The core module, CSPLayer, contains a large convolutional kernel that enables the detection network to capture contextual information more comprehensively and accurately. In addition, a universal convolutional layer has been added to the backbone layer to effectively extract more valuable information from the image. Then, a BoT3 module with a multi-head self-attention (MHSA) mechanism was added to the neck module of YOLOv5, which improved the detection network’s performance in target dense scenes and further enhanced the detection accuracy.

However, these network design strategies utilized in the studies were effective towards detection of very small targets, which satisfied that task, and may not be suitable for detection of mask and nose targets since the focus on extremely small targets can lead to misrecognition of masks and noses. Excessive focus on detecting small-sized targets can reduce the generalization performance of a trained model. On the other hand, the target objects that need to be identified in our study are two types, and model design strategies with too many layers and large parameter quantities or volumes cannot meet the requirements of algorithm design for real-time monitoring systems in this study.

### 3.4. Comparative Discussion on Performance of the Model Proposed in the Study

Furthermore, an image editing network and three types of masked face detection datasets (the Correctly Masked Face Dataset (CMFD), the Incorrectly Masked Face Dataset (IMFD) and their combination (MaskedFace-Net) for the masked face detection) were proposed in this study [28]. The function of the network proposed in this study is to generate a dataset for mask wearing detection. Specifically, the network is used to generate a dataset of mask images covering different positions on people’s face. Although the network involves matching and detecting feature points such as the nose on facial images, it is not yet a dedicated network model for detecting mask wearing standardization. Therefore, this network cannot be directly applied to detect whether the behavior of people wearing masks is standardized in real scenes.

On the other hand, due to the involvement of multiple feature point matching functions in the proposed model in [28], the size of the model will be large, and the applicability of embedding it into the hardware of medical detection systems for real-time detection is unknown. However, the network model proposed in our study is lightweight, with a model size of only 12.6 MB and a detection speed of 71 fps. It is specifically designed for real-time detection of mask wearing standardization task.

Comparing the performance of the algorithm proposed in this study with other mask wearing normalization detection algorithms, Table 8 shows that the detection accuracy of the proposed model is relatively high, only slightly lower than the one proposed in study [19] (0.2%). However, compared with the algorithm proposed in that study, the detection speed of the algorithm designed in our article is 0.05 s/pic (55fps) faster. The algorithm proposed in this study has a significantly faster detection speed, resulting in better overall performance and better suitability for embedding in hardware devices of medical real-time monitoring systems for mask wearing normalization detection, while satisfying the actual application detection accuracy. On the other hand, the size of the model proposed in this study is only 12.6 MB, which is slightly higher than size of the model proposed in reference [14] (11 MB). However, the detection accuracy of the model designed in our article is higher, and the detection speed is 10 times faster than the model proposed in reference [14], thus indicating the advantages in overall performance of our detection model.

## 4. Conclusions

The deep learning detection model proposed in this study is an improved design model based on the YOLOv5s model, which is the essence of the proposed method. In the main text, Section 2.1.1 elaborates on the structure of the original YOLOv5s model, while Section 2.1.2, Section 2.1.3, and Section 2.1.4, respectively, describe our improvement operations on the YOLOv5s model. Among them, Section 2.1.2 describes the improvement of the YOLOv5s model backbone network, Section 2.1.3 describes the embedding of a feature extraction module (SE module) in the YOLOv5s model that can improve detection accuracy, and Section 2.1.4 describes our improvement method for the cross-connection between different feature extraction layers in the YOLOv5s model.

(1) Aiming at the problem that most existing mask detection algorithms only detect whether people are wearing a mask and cannot detect the situation of ‘non-normalized wearing’, a mask wearing normalization detection algorithm based on improved YOLOv5s was proposed, which achieved mask wearing normalization detection for different persons in complex backgrounds. This study can provide technical support for the development of intelligent medical detection systems and ensuring the safety and health of people in the post-pandemic era.
(2) The BottleneckCSP module was improved to a BottleneckCSP-MASK module, achieving lightweight improvement of the backbone network for the detection model. An SE module was inserted to the proposed improved backbone network, and the bonding fusion layer of the model was improved for better extracting the features of mask and nose targets.
(3) The experimental results indicated that, for different people and complex backgrounds, the improved YOLOv5s-MASK model could effectively recognize the behaviors of people, including not wearing a mask, normalized wearing, and wearing a mask non-normalized. The overall detection accuracy was 99.3%, with an average detection speed of 0.014 s/pic.
(4) Contrasted with original YOLOv5s, v5m, and v5l models, the detection results for the two types of target objects in the test set indicated that the mAP of the designed YOLOv5s-MASK network increased by 0.5%, 0.49%, and 0.52%, respectively, and the size of the proposed model compressed to 90% of the original v5s model.

## 5. Future Work

Although the proposed algorithm has a high detection accuracy and can meet the requirements of real-time accurate detection, the training images were all generated during the day, so the algorithm is not suitable for detecting mask wearing in environments with weak nighttime light intensity. This is the limitation of the proposed algorithm. On the other hand, the number of masks with different appearance types included in model training is limited. In order to make the model more applicable, it is necessary to add more diverse mask image data with different appearance styles to the training dataset at a later stage. The proposed detection model in this study improved only towards the mask wearing normalization recognition in daytime, and can therefore be utilized in illuminated environments. Although many detection places are equipped with lighting installations, to further expand the applicability of the proposed algorithm, image data for mask wearing normalization detection captured by night vision cameras (including night vision monitors) will be added to the training sets which are used for training of the detection model, in order to realize automatic recognition for behaviors of people at places without lighting equipment during day and night, including not wearing a mask, normalized wearing, and wearing a mask non-normalized. On the other hand, at a later stage, feature extraction module in the backbone network of detection model can be replaced with other advanced modules with residual structures in future work, in order to achieve further optimization and improvement of model detection accuracy and reduce model weight. Furthermore, the recognition of target objects for mask wearing normalization in images captured by cameras equipped in unmanned aerial vehicles [29,30,31,32] utilizing the detection network architecture proposed in this study is also worth studying in the future.

## Figures and Tables

**Table 1:** Research on deep learning-based mask target detection.

Detection Networks	Accuracy (%)	Year	Whether They Detect Mask Wearing Normalization	Reference
SCRNet	98.7	2020	N	[7]
SSD + MobileNetV2	91.7	2020	N	[8]
ResNet-50 + YOLOv2	81	2021	N	[10]
SSDMNV2	92	2021	N	[9]
VGG-16	96	2020	N	[13]
MobileNetV2	97.81	2023	Y	[14]
ResNet50 + SVM	99.64	2021	N	[11]
Improved YOLOv3	99.5	2021	Y	[19]
Transfer learning + Efficient-Yolov3	96.03	2022	N	[15]
VGG-19	99	2021	N	[16]
VGG16	84	2021	Y	[12]
YOLOv4	73.8	2023	Y	[18]
MobileNetV2	92.64	2021	N	[9]
Fast RCNN + InceptionV2	91.1	2021	Y	[17]

Y: Yes; N: No.

**Table 2:** Model parameters of four YOLOv5 architectures.

Model	Depth	Width	Layer	Parameters
YOLOv5s	0.33	0.50	191	7.26 × 10^6^
YOLOv5m	0.67	0.75	263	2.15 × 10^7^
YOLOv5l	1.0	1.0	335	4.78 × 10^7^
YOLOv5x	1.33	1.25	407	8.90 × 10^7^

**Table 3:** Amount distribution of different classes in training and test sets.

	Image Data Set	Training Set	Test Set
Mask Wearing Styles			
Normalized wearing		452	50
Non-normalized wearing		466	50
No mask		548	50

**Table 4:** Recognition results for mask and nose targets based on YOLOv5s-MASK model.

Class	Number	Precision (%)	Recall (%)	mAP (%)
Total	201	98	99	99
Nose	101	98	98	98.5
Mask	100	98	100	99.5

**Table 5:** Detection results of mask wearing normalization based on YOLOv5s-MASK model.

Class	Number	Correctly Detected Number	*P_mask_* (%)
Total	150	149	99.3
Normalized wearing	51	51	100
Non-normalized wearing	49	49	100
No mask	50	49	98

**Table 6:** Network performance of different models.

Target Detection Networks	mAP (%)
YOLOv5s	98.49
YOLOv5s + SE	98.56
YOLOv5s + BottleneckCSP-MASK	98.62
YOLOv5s + BottleneckCSP-MASK + SE	98.99

**Table 7:** Performance comparison of various detection models.

Target Detection Networks	mAP (%)	Average Detecting Time (s/pic)	Number of Parameters	Size of Models (MB)
YOLOv5s	98.49	0.013 (76.9 fps)	7.3 × 10^6^	14.0
YOLOv5m	98.50	0.071 (14 fps)	2.2 × 10^7^	41.3
YOLOv5l	98.47	0.076 (13 fps)	4.8 × 10^7^	90.8
YOLOv5s-MASK	98.99	0.014 (71 fps)	6.5 × 10^6^	12.6

**Table 8:** Research on deep learning-based mask wearing normalization detection.

Detection Networks	Accuracy (%)	Model Size (MB)	Average Detecting Time (s/pic)	Year	Reference
MobileNetV2	97.81	11	0.14 (7 fps)	2023	[14]
Improved YOLOv3	99.5	—	0.064 (16 fps)	2021	[19]
VGG16	84	528	—	2021	[12]
YOLOv4	73.8	—	>0.02 (<50 fps)	2023	[18]
Fast RCNN + InceptionV2	91.1	—	0.073 (14 fps)	2021	[17]
Ours (YOLOv5s-MASK)	99.3	12.6	0.014 (71 fps)	2024	—

## Data Availability

The original contributions presented in the study are included in the article, further inquiries can be directed to the corresponding author.
